# Recent Advances in CNS P2X7 Physiology and Pharmacology: Focus on Neuropsychiatric Disorders

**DOI:** 10.3389/fphar.2018.00030

**Published:** 2018-02-01

**Authors:** Anindya Bhattacharya

**Affiliations:** Neuroscience Therapeutic Area, Janssen Research and Development, LLC, San Diego, CA, United States

**Keywords:** P2X7, microglia, depression, IL-1beta, schizophrenia, bipolar disorder, neuroinflammation, neuropsychiatry

## Abstract

The ATP-gated P2X7 ion channel is an abundant microglial protein in the CNS that plays an important pathological role in executing ATP-driven danger signal transduction. Emerging data has generated scientific interest and excitement around targeting the P2X7 ion channel as a potential drug target for CNS disorders. Over the past years, a wealth of data has been published on CNS P2X7 biology, in particular the role of P2X7 in microglial cells, and *in vivo* effects of brain-penetrant P2X7 antagonists. Likewise, significant progress has been made around the medicinal chemistry of CNS P2X7 ligands, as antagonists for *in vivo* target validation in models of CNS diseases, to identification of two clinical compounds (JNJ-54175446 and JNJ-55308942) and finally, discovery of P2X7 PET ligands. This review is an attempt to bring together the current understanding of P2X7 in the CNS with a focus on P2X7 as a drug target in neuropsychiatric disorders.

## Introduction

P2X7 is an ATP-gated ion channel; activation of the ion channel by ATP leads to opening of the pore causing non-selective flux of Ca^2+^, Na^+^, and K^+^ ions (Bartlett et al., 2014). In addition to the non-selective cation conductance, P2X7 channel opening under constant stimulation to ATP is believed to form a “macro-pore,” the function of which has remained elusive; pharmacologically, there is no antagonist or modulator of P2X7 that selectively disrupts the macro-pore and as such the physiological relevance of the macro-pore has been debated and recently challenged (Pippel et al., 2017). In addition to ion flux, the best studied downstream function of P2X7 is the release of pro-inflammatory cytokines, IL-1β and IL-18 (He et al., 2017) that functions through the recruitment of the NLRP3 inflammasome complex (Giuliani et al., 2017). Since P2X7 is abundantly expressed in blood cells, IL-1β release in the blood has been used as a biomarker of P2X7 activity and has been used in both preclinical and clinical assessment of target engagement (Stock et al., 2012; Letavic et al., 2017). Although the P2X7-dependent NLRP3-mediated IL-1β release is the best characterized signature of P2X7 activity *in vivo*, there are numerous other physiological endpoints (pyroptosis, membrane blebbing shedding of cell surface proteins, microvesicle/exosome release to even modulation of synaptic events), some or all of which may contribute to P2X7-driven pathology (Bartlett et al., 2014). P2X7 function is even modulated by cholesterol and other membrane lipids (Karasawa et al., 2017). Within the CNS P2X7 is abundantly expressed in microglial cells (Bhattacharya and Biber, 2016) and P2X7 activation causes IL-1β (and IL-18) release, neuroinflammation, and microglial activation (Monif et al., 2010; Bhattacharya et al., 2013; He et al., 2017). In addition to microglia, astrocytes and oligodendrocytes also express this ion channel subtype in the CNS; the expression of P2X7 in CNS neurons is inconsistent and sometimes controversial (Illes et al., 2017). Even though P2X7 expression is abundant in microglia (and in peripheral immune cells), the channel is “silent” under normal physiology where ATP concentrations do not reach high micromolar levels to activate the ion channel (Bhattacharya and Biber, 2016). As such, from a drug discovery perspective, P2X7 is an ideal drug target as antagonism of a silent channel by true neutral antagonists would not cause any serious target mediated (adverse) effects, i.e., antagonism will only elicit when the channel is activated by high ATP concentrations (i.e., on-demand activation, danger signal), which is believed to be achieved during pathology of neuroinflammatory disorders of the CNS such as stroke, epilepsy, multiple sclerosis, chronic neurodegenerative, and neuropsychiatric diseases. There have been several excellent and comprehensive review articles on P2X7 highlighting the biology, genetics, and disease relevance (Bartlett et al., 2014; Sperlagh and Illes, 2014; De Marchi et al., 2016; Park and Kim, 2017); for this mini-review, the focus is entirely on the role of CNS P2X7 in neuropsychiatry, with an emphasis on pharmacology and medicinal chemistry of brain-penetrant P2X7 antagonists including the identification of the two CNS P2X7 clinical compounds, JNJ-54175446 and JNJ-55308942 (see later).

## Role of P2X7 in Neuropsychiatric Disorders

Chronic inflammation is one of the core underpinnings of many diseases; relatively recent scientific literature over the past few years points to the role of peripheral and central inflammation in neuropsychiatric diseases (Leighton et al., 2017; Wetsman, 2017). With the relatively recent discovery of CNS lymphatics in the meningeal tissue that drains CSF into deep cervical nodes (Louveau et al., 2015), and emerging science supporting the possibility of compromised blood–brain, blood–CSF, blood–choroid plexus barriers in chronic diseases of the CNS, the role of immune cells and their signaling partners in CNS pathophysiology are beginning to be thoroughly appreciated (Wohleb et al., 2016). As has happened in the space of immuno-oncology, there is a now a growing body of scientific literature that supports a role of immunology in neuropsychiatry, the emerging discipline of neuroimmunopsychiatry (Bhattacharya et al., 2016). Perhaps, it is not a mere coincidence that several inflammatory disorders are co-morbid with depression, bipolar disorder, and schizophrenia (Miller et al., 2017). This has been supported by immune-related gene enrichment in patients (Jansen et al., 2016; Leday et al., 2018).

Within the CNS, microglia are considered as innate immune cells and one of consequences of activated microglia results in neuroinflammation as defined by the release of several pro-inflammatory cytokines including IL-1β. This cytokine is present at higher levels in plasma, CSF, and postmortem brain tissue of individuals with mood disorders (Söderlund et al., 2011; Jones and Thomsen, 2013). This cytokine has been linked with geriatric depression and postpartum depression as well (Corwin et al., 2008; Diniz et al., 2010). In animal models of stress-induced depression, it was shown that IL-1β signaling was critical to the development of depression-like phenotype (Koo and Duman, 2009). Due to this body of data it is reasonable to hypothesize that targeting P2X7, upstream of NLRP3 and IL-1β signaling, with CNS penetrable P2X7 antagonists would be beneficial for treating mood disorders. As such, there is a growing body of literature that strengthens the role of P2X7-IL-1β pathway in mood disorders including depression and bipolar disorder (Chrovian et al., 2014; Stokes et al., 2015; Bhattacharya and Biber, 2016; Bhattacharya and Drevets, 2017). Some of the key data supporting the role of P2X7 in models of depression, mania, and schizophrenia is discussed below.

P2X7 activation produces microglial activation (Monif et al., 2010). Microglial activation has been clinically demonstrated in depression, bipolar disorder, and schizophrenia by use of PET ligands designed to target TSPO, a mitochondrial protein used as a surrogate of microglial activation (Mondelli et al., 2017). As such, it is plausible that P2X7 plays a role in the etiology of neuropsychiatric diseases, maybe in a subset of patients with neuroinflammation. Recently, P2X7 has come to the light as a potential molecular player in schizophrenia (Kovanyi et al., 2016). This is not surprising given the role of microglia and neuroinflammation in schizophrenia (Laskaris et al., 2016). For depression and bipolar disorder, there is even more growing genetic evidence of P2X7, although the penetrance and frequency of the SNPs to disease susceptibility and protection is not clearly understood. It is perhaps not surprising that the data is equivocal, clouded with both positive and negative associations (Barden et al., 2006; Lucae et al., 2006; Hejjas et al., 2009; Backlund et al., 2011, 2012; Soronen et al., 2011; Halmai et al., 2013). One of the most reported P2X7 SNP is rs2230912. The rs2230912-G allele is known to exhibit a gain-of-function for IL-1β release from human monocytes (Stokes et al., 2010). A recent meta-analysis was reported for rs2230912 and the authors concluded strong association of rs2230912 with depression and bipolar disorder (Czamara et al., 2017), although negative association meta-analysis also exists for this particular SNP (Feng et al., 2014). In addition to rs2230912, there are additional SNPs on the *p2rx7* gene (rs1718119, rs3751143, rs1653624) that may shed additional insight into the role of P2X7 SNPs and disease susceptibility; perhaps, more detailed haplotype analysis is needed to understand the relationship between allelic variation, function (IL-1β release), and disease protection and/or susceptibility. In addition to the human genetic literature, emerging science in animal models of despair and anhedonia has been supportive of the P2X7 hypothesis of mood disorders. Several groups have demonstrated an anti-depressant and anti-manic phenotype of P2X7 knockout mice (Basso et al., 2009; Boucher et al., 2011; Csolle et al., 2013a,b; Wilkinson et al., 2014). Whether the knockout phenotypes, in particular the acute despair like behaviors seen in forced swim immobility measurements, can be robustly recapitulated in rodents with pharmacological specificity remains to be seen as it is not clear how ATP would activate central P2X7 channels in an acute stressful setting. Where P2X7 probably plays a more significant role is in chronic settings of stress, where IL-1β-driven microglial activation and neuroinflammation has been shown to upregulate and P2X7 antagonism may be efficacious. In line with this hypothesis, in a model of sucrose consumption that is reflective of hedonic behavior, pharmacological antagonism of P2X7 restored the deficit observed in drinking sucrose-water (anhedonia) either under chronic stress or by systemic administration of lipopolysaccharides (LPS) (Csolle et al., 2013b). Consistent with these observations, recent data with P2X7 selective, brain-penetrant antagonists demonstrated efficacy in chronic models of stress (Lovenberg et al., 2015; Iwata et al., 2016; Yue et al., 2017); these findings point to a pathway of stress mediated ATP-driven activation of P2X7–NLRP3–IL-1β pathway, leading to microglial activation (pro-inflammatory) and neuroinflammation. A recent study demonstrated enhanced IL-1β release in the brain, upregulation of P2X7 mRNA, and microglial activation in a chronic stress paradigm (Tan et al., 2017). Chronic stress is known to contribute to clinical depression (Calcia et al., 2016) and as such there is hope that P2X7 antagonists with good CNS penetration and drug likeliness will proceed into clinical testing as novel mechanisms for mood disorders. There is also a recent publication indicating the role of P2X7 channels in modulating stress-mediated spine density downregulation and P2X7 knock out mice are protective from this decrease in spine density (Otrokocsi et al., 2017). In addition to depression, blockade of P2X7 may be useful as mood stabilizer in bipolar disorder (Gubert et al., 2014). P2X7 antagonism was efficacious in amphetamine-induced sensitization of hyperactivity (Bhattacharya et al., 2013; Lord et al., 2014), and similar phenotypes were observed in P2X7 knockout mice (Gubert et al., 2014). Taken together, the body of emerging data suggests a potential therapeutic utility of brain-penetrant P2X7 antagonists in mood disorders, especially targeting treatment resistant patient sub-populations or as an adjunct to current pharmacotherapy for efficacy maintenance.

## P2X7 Pharmacology: Brain-Penetrant Antagonists

**FIGURE 1:**
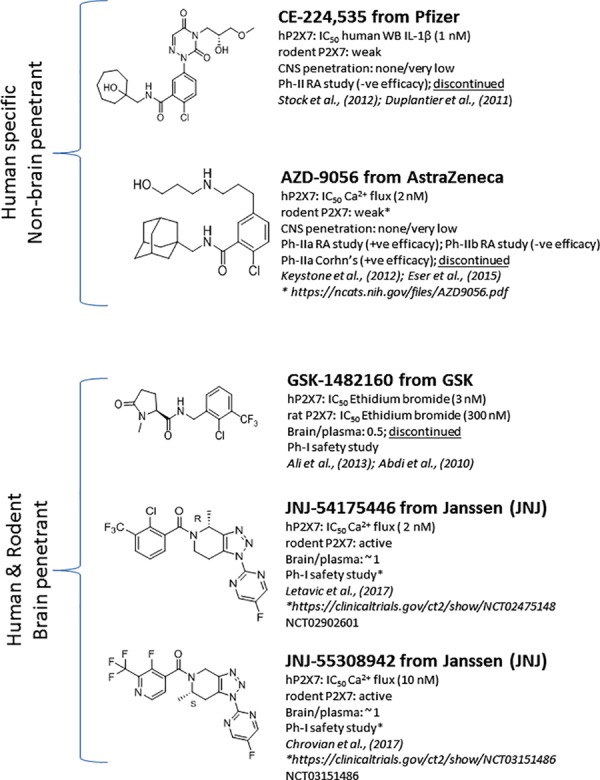
P2X7 antagonists that transitioned into clinical development.

**FIGURE 2:**
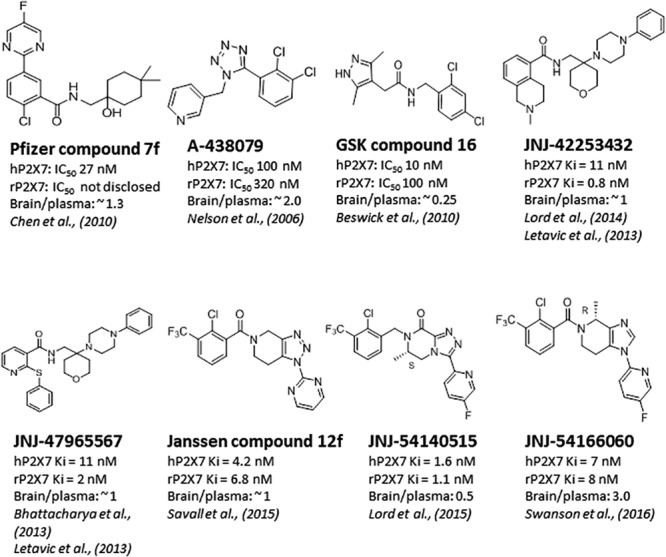
Brain-penetrant P2X7 antagonists.

Significant progress has been made toward identification of brain-penetrant P2X7 antagonists. This spans medicinal chemistry efforts from identification of tool molecules to selection of brain-penetrant clinical candidates JNJ-54175446 (Letavic et al., 2017) and JNJ-55308942 (Chrovian et al., 2017). Unlike the Pfizer and AstraZeneca clinical compounds (**Figure 1**), the Janssen molecules retain rodent activity providing the discovery team to develop robust target engagement assays to drive the chemistry program; in addition, rodent activity provided the team with an opportunity to test the molecules in rodent models of disease, an important missing link in the prior two clinical compounds (CE-224,535 and AZD-9056). Medicinal chemistry efforts toward identification of brain-penetrant P2X7 ligands in the industry are summarized in **Figure 2**. The Pfizer molecule (compound 7f) was reported to be drug like with a low clearance, long half-life in rats, and good CNS exposure (brain/plasma of 1.3) (Chen et al., 2010). Medicinal chemistry groups at Abbott Laboratories (now Abbvie) and GlaxoSmithKline (GSK) were the pioneers in discovering scaffolds with both rodent potency and low-moderate CNS permeability (Nelson et al., 2006; Beswick et al., 2010; Chen et al., 2010). The group at Janssen has published several papers disclosing P2X7 antagonists with excellent brain penetration (**Figure 2**) and focusing on brain P2X7-mediated pharmacodynamic endpoints (Bhattacharya et al., 2013; Letavic et al., 2013; Lord et al., 2014; Savall et al., 2015). JNJ-47965567 and JNJ-42253432 demonstrated activity at rodent and human P2X7, had good rat pharmacokinetic profiles, and excellent brain penetration when dosed subcutaneously to allow for >80% brain P2X7 occupancy, as judged by *ex vivo* autoradiography in rat brain slices. Docking studies with JNJ-47965567 reveal an allosteric binding site (Karasawa and Kawate, 2016). More recently, Janssen disclosed several additional classes of P2X7 antagonists, beginning with a series of fused 1,2,3-triazoles. A closer look at the nuances of adding the methyl substituents to the 1,2,4-triazolopyrazine core revealed a strong enantiomeric preference of the P2X7 ion channel. Chiral separation and ultimately enantioselective synthesis demonstrated the (S)-7-methyl derivative (human IC_50_ of 7.7 ± 2.6 nM and rat IC_50_ of 8.0 ± 2.9 nM) was highly favored over the (R)-8-methyl derivative devoid of any significant P2X7 activity (human IC_50_ of 1560 nM and rat IC_50_ of 2240 nM). JNJ-54140515 was discovered from this series and was shown to readily cross the blood–brain barrier facilitating the high level of brain P2X7 occupancy. In addition to good *in vivo* properties, this ligand was highly selective and was tritiated to produce a radioligand for P2X7 (Lord et al., 2015). JNJ-54166060 was discovered from an imidazopyridine scaffold (Swanson et al., 2016) and was shown to have a significant brain/plasma ratio of close to 3. Although the brain concentrations were approximately threefold greater than plasma levels, free plasma and free brain concentrations were similar after correcting for brain binding (1.5% free) and plasma protein binding (5.5% free). The compound demonstrated an ED_50_ of 2.3 mg/kg oral dose.

The discovery of the clinical compound JNJ-54175446 (**Figure 1**; NCT02902601) and JNJ-55308942 (**Figure 1**; NCT03151486) is described in detail by Letavic et al. (2017) and Chrovian et al. (2017). JNJ-54175446 is a high affinity (and potency) P2X7 antagonist that displays pharmacology at recombinant human, rat, mouse, macaque, and dog P2X7. In native systems, this compound displayed P2X7 pharmacology as well: it blocked P2X7-dependent IL-1β release in human blood and displayed binding to rat, dog, and human brain sections. Target engagement in rat brain was demonstrated for JNJ-54175446 utilizing *ex vivo* autoradiography. The molecule showed dose-dependent brain P2X7 occupancy with an ED_50_ of 0.46 mg/kg, which corresponded to plasma EC_50_ of 105 ng/ml. This compound efficiently crossed into the brain compartment with a brain/plasma ratio of approximately 1.1 in rat. JNJ-54175446 demonstrated robust and sustained P2X7 brain occupancy in the rat following a 10 mg/kg oral dose (>80% occupancy for 24 h). Consistent with rat brain target engagement, the compound did attenuate brain IL-1β release, as measured by microdialysis. When the molecule was dosed in a higher species (i.e., dog), JNJ-54175446 also bound to dog brain P2X7, and blocked dog blood IL-1β release, providing a model of both central and peripheral target engagement in one species. This data highlights the fact that blood IL-1β can be used as a biomarker, and may be a surrogate for central target engagement in the absence of a PET ligand. As has been described later on, there are several P2X7 PET ligands that should enable human target engagement (i.e., human brain P2X7) studies. Given the excellent target engagement data in rats and dogs, JNJ-54175446 was profiled further to enrich clinical candidacy data package including toxicity and cardiovascular safety studies. JNJ-55308942, has been chosen as the second P2X7 antagonist from Janssen to enter clinical trials, as a back-up to JNJ-54175446. While maintaining the superior P2X7 pharmacology of the lead molecule, JNJ-55308942 provides significant improvement in solubility and protein binding properties (i.e., increased free fraction). JNJ-55308942 showed excellent P2X7 receptor occupancy in the hippocampus of rats, demonstrating a low ED_50_ of 0.07 mg/kg. The compound also demonstrated good tolerability margins in preclinical species, as well as an acceptable cardiovascular safety profile *in vivo*.

In addition to Janssen’s efforts, several other companies (Affectis, Axxam, Actelion, Lundbeck) have published patents on brain-penetrant P2X7 scaffolds (Park and Kim, 2017). Both Lundbeck and Axxam Pharmaceuticals published patents disclosing CNS-penetrant P2X7 antagonists, with Axxam entering into a collaborative agreement with Alzheimer’s drug discovery foundation to understand the role of centrally penetrant P2X7 antagonists in Alzheimer’s. Lastly, AFC-5128 is a brain-penetrant P2X7 antagonist from Affectis Pharmaceuticals that is being pushed for neuropathic pain and multiple sclerosis indications (company website).

## P2X7 Pet Ligands

**FIGURE 3:**
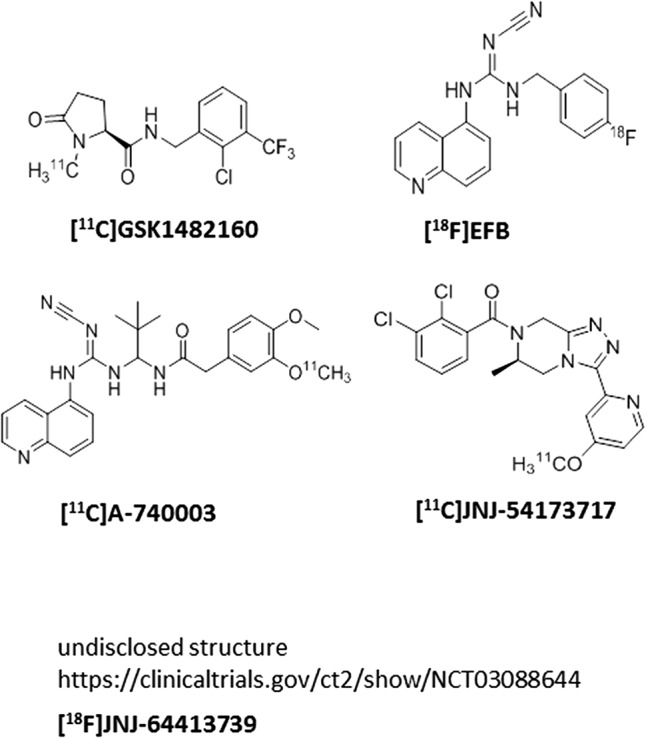
P2X7 PET ligands.

Several P2X7 PET ligands have been described in the literature recently (**Figure 3**); there are two clinical utilities of P2X7 PET ligands that cross the blood–brain barrier: (a) the PET ligands can be used as a probe to measure central target occupancy of the clinical compound being developed for a CNS indication; (b) since P2X7 activation is related to microglial activation, P2X7 PET ligands can potentially be used as central biomarkers of assessing microglial activation in diseases accompanying neuroinflammation such as mood disorders, schizophrenia, epilepsy, multiple sclerosis, and others. Our team has disclosed a [^11^C]-labeled P2X7 PET ligand (JNJ-54173717), where it was shown that P2X7 overexpression can be detected under basal and overexpressed conditions and the PET signal was blocked by competition with cold P2X7 ligands in a dose-dependent manner in rats (Ory et al., 2016). A second P2X7 PET ligand, with [^18^F] label (JNJ-64413739), has been recently described at the American College of Neuropsychopharmacology conference (December, 2017) where it was elegantly demonstrated that JNJ-54175446 (i.e., the clinical compound shown in **Figure 1**) was able to displace [^18^F]JNJ-64413739 PET signal in healthy human subjects in a dose-dependent manner (NCT03088644). Similarly, the [^11^C]GSK1482160 PET signal in the brain of LPS challenged rats was also blocked by a cold P2X7 antagonist, demonstrating specificity of the PET signal to P2X7 (Territo et al., 2017). In addition to [^18^F]JNJ-64413739, [^11^C]JNJ-54173717, and [11C]GSK1482160, two other groups have disclosed P2X7 PET ligands ([^11^C]A-7400003 and [^18^F]-EFB), albeit with no data to support P2X7 specificity of the brain signal (Janssen et al., 2014; Fantoni et al., 2017). The field has made significant progress in discovery of brain-penetrant P2X7 antagonists in recent years. Molecules such as JNJ-54175446 will now need to be tested in clinical settings to find efficacy signals in psychiatric disorders; persistent and prudent drug developmental paradigms, out-of-box thinking, challenging the status-quo, may result in a P2X7 therapeutic that will benefit patients suffering from life-changing CNS disorders.

## Conclusion and Perspectives

The unmet medical need in neuropsychiatry is staggering; it is clear from existing clinical data that “one pill fits all” strategy has not worked. Patients suffering from depression, bipolar disorder, and schizophrenia may have neuroinflammatory causality of the disease, and not every patient carries similar inflammatory disease burden. While there are patients that will benefit from existing pharmacotherapy, a huge sub-population of patients will respond better to therapies that address the underlying cause of the disease and the hope is CNS P2X7 antagonists in clinical trials will advance into proof of concept studies either as monotherapy or as adjunctives for neuropsychiatric disorders.

## Author Contributions

AB conceived the topic for the mini-review and wrote the draft of the manuscript.

## Conflict of Interest Statement

The author is a full-time employee of Johnson & Johnson.
